# Dibromidobis(pyrazine-2-carboxamide-κ*N* ^4^)zinc

**DOI:** 10.1107/S1600536812013335

**Published:** 2012-03-31

**Authors:** Sadif A. Shirvan, Sara Haydari Dezfuli

**Affiliations:** a Department of Chemistry, Omidieh Branch, Islamic Azad University, Omidieh, Iran

## Abstract

The title complex, [ZnBr_2_(C_5_H_5_N_3_O)_2_], shows crystallographic mirror symmetry with the Zn atom and the two bromine ligands located on the mirror plane. The Zn atom is four-coordinated in a distorted tetra­hedral fashion by two N atoms from two pyrazine-2-carboxamide ligands and two Br atoms. Only one of the amino H atoms is involved in an N—H⋯O hydrogen bond. The crystal packing is further stabilized by weak N—H⋯N and C—H⋯O inter­actions.

## Related literature

For related structures, see: Abu-Youssef *et al.* (2006 ▶); Azhdari Tehrani *et al.* (2010 ▶); Goher & Mautner (2000 ▶); Kristiansson (2002 ▶); Mir Mohammad Sadegh *et al.* (2010 ▶); Munakata *et al.* (1997 ▶); Pacigova *et al.* (2008 ▶).
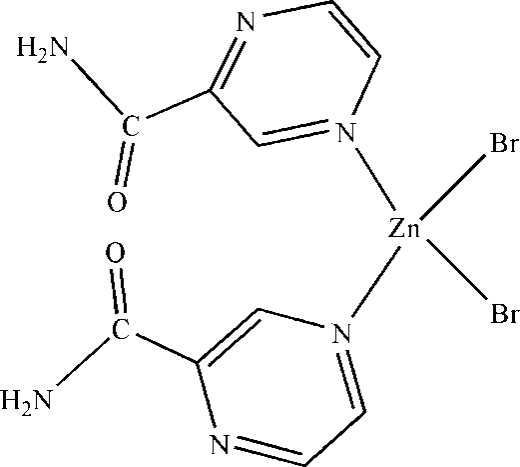


## Experimental

### 

#### Crystal data

[ZnBr_2_(C_5_H_5_N_3_O)_2_]
*M* *_r_* = 471.43
Monoclinic, 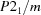

*a* = 5.6042 (4) Å
*b* = 19.5147 (19) Å
*c* = 7.0656 (5) Å
β = 106.835 (5)°
*V* = 739.61 (10) Å^3^
*Z* = 2
Mo *K*α radiation
μ = 7.08 mm^−1^
*T* = 298 K
0.25 × 0.24 × 0.20 mm

#### Data collection

Bruker APEXII CCD area-detector diffractometer
Absorption correction: multi-scan (*SADABS*; Bruker, 2007 ▶) *T*_min_ = 0.205, *T*_max_ = 0.250
3935 measured reflections
1495 independent reflections
1247 reflections with *I* > 2σ(*I*)
*R*_int_ = 0.053

#### Refinement

*R*[*F* ^2^ > 2σ(*F* ^2^)] = 0.046
*wR*(*F* ^2^) = 0.111
*S* = 1.10
1495 reflections
100 parameters
H-atom parameters constrained
Δρ_max_ = 0.77 e Å^−3^
Δρ_min_ = −1.19 e Å^−3^

### 

Data collection: *APEX2* (Bruker, 2007 ▶); cell refinement: *SAINT* (Bruker, 2007 ▶); data reduction: *SAINT*; program(s) used to solve structure: *SHELXS97* (Sheldrick, 2008 ▶); program(s) used to refine structure: *SHELXL97* (Sheldrick, 2008 ▶); molecular graphics: *SHELXTL* (Sheldrick, 2008 ▶); software used to prepare material for publication: *SHELXTL*.

## Supplementary Material

Crystal structure: contains datablock(s) I, global. DOI: 10.1107/S1600536812013335/bt5863sup1.cif

Structure factors: contains datablock(s) I. DOI: 10.1107/S1600536812013335/bt5863Isup2.hkl

Additional supplementary materials:  crystallographic information; 3D view; checkCIF report

## Figures and Tables

**Table 1:** Hydrogen-bond geometry (Å, °)

*D*—H⋯*A*	*D*—H	H⋯*A*	*D*⋯*A*	*D*—H⋯*A*
N3—H3*B*⋯O1^i^	0.86	2.01	2.869 (7)	175
N3—H3*C*⋯N2	0.86	2.38	2.732 (7)	105
N3—H3*C*⋯N2^ii^	0.86	2.54	3.182 (7)	132
C3—H3⋯O1^iii^	0.93	2.49	3.414 (7)	172

Symmetry codes: (i) 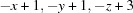; (ii) 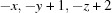; (iii) 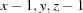.

**Fig. 1.**
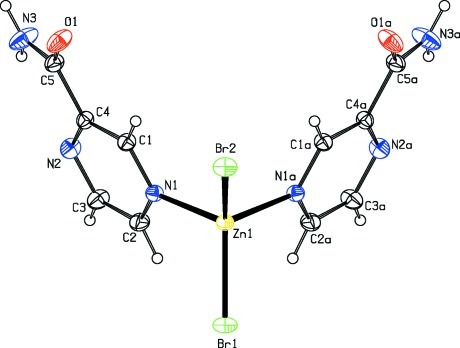
The molecular structure of the title molecule, with the atom-numbering scheme. Displacement ellipsoids are drawn at the 30% probability level. [Symmetry codes: (a) x,3/2 - y,z].

**Fig. 2.**
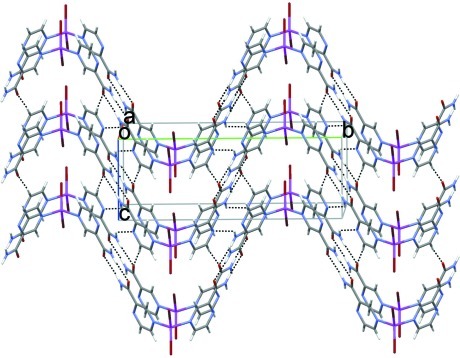
Unit-cell packing diagram for title molecule. Hydrogen bonds are shown as dashed lines.

## supplementary crystallographic information

### Comment

A few complexes of pyrazine-2-carboxamide (pzc) have been prepared, such as that of mercury (Azhdari Tehrani *et al.*, 2010; Mir Mohammad Sadegh *et al.*, 2010) and vanadium (Pacigova *et al.*, 2008), manganese (Abu-Youssef *et al.*, 2006) and copper (Kristiansson, 2002; Munakata *et al.*, 1997; Goher & Mautner, 2000).

The asymmetric unit of the title compound, (Fig. 1), contains one Zn^II^ atom, two Br atoms and one pyrazine-2-carboxamide ligand. The Zn^II^ atom is four-coordinated in a distorted tetrahedral configuration by two N atoms from two pyrazine-2-carboxamide ligands and two terminal Br atoms.

Only one of the amino H atoms is involved in a N—H···O hydrogen bond. The crystal packing is further stabilized by weak N—H···N and C—H···O interactions.

### Experimental

A solution of pyrazine-2-carboxamide (0.25 g, 2.0 mmol) in methanol (10 ml) was added to a solution of ZnBr_2_ (0.23 g, 1.0 mmol) in methanol (10 ml) and the resulting colourless solution was stirred for 15 min at room temperature. This solution was left to evaporate slowly at room temperature. After one week, colourless block shaped crystals of the title compound were isolated (yield 0.38 g, 80.6%).

### Refinement

All H atoms were positioned geometrically, with C—H = 0.93 Å and N—H = 0.86 Å and constrained to ride on their parent atoms, with *U*_iso_(H)=1.2*U*_eq_(C,N).

### Crystal data

**Table:**

[ZnBr_2_(C_5_H_5_N_3_O)_2_]	*F*(000) = 456
*M**_r_* = 471.43	*D*_x_ = 2.117 Mg m^−^^3^
Monoclinic, *P*2_1_/*m*	Mo *K*α radiation, λ = 0.71073 Å
*a* = 5.6042 (4) Å	Cell parameters from 3935 reflections
*b* = 19.5147 (19) Å	θ = 3.0–26.0°
*c* = 7.0656 (5) Å	µ = 7.08 mm^−^^1^
β = 106.835 (5)°	*T* = 298 K
*V* = 739.61 (10) Å^3^	Block, colorless
*Z* = 2	0.25 × 0.24 × 0.20 mm

### Data collection

**Table:**

Bruker APEXII CCD area-detector diffractometer	1495 independent reflections
Radiation source: fine-focus sealed tube	1247 reflections with *I* > 2σ(*I*)
Graphite monochromator	*R*_int_ = 0.053
ω scans	θ_max_ = 26.0°, θ_min_ = 3.0°
Absorption correction: multi-scan (*SADABS*; Bruker, 2007)	*h* = −6→6
*T*_min_ = 0.205, *T*_max_ = 0.250	*k* = −21→24
3935 measured reflections	*l* = −8→8

### Refinement

**Table:**

Refinement on *F*^2^	Primary atom site location: structure-invariant direct methods
Least-squares matrix: full	Secondary atom site location: difference Fourier map
*R*[*F*^2^ > 2σ(*F*^2^)] = 0.046	Hydrogen site location: inferred from neighbouring sites
*wR*(*F*^2^) = 0.111	H-atom parameters constrained
*S* = 1.10	*w* = 1/[σ^2^(*F*_o_^2^) + (0.0663*P*)^2^] where *P* = (*F*_o_^2^ + 2*F*_c_^2^)/3
1495 reflections	(Δ/σ)_max_ = 0.003
100 parameters	Δρ_max_ = 0.77 e Å^−^^3^
0 restraints	Δρ_min_ = −1.19 e Å^−^^3^

### Special details

**Table:**

Geometry. All e.s.d.'s (except the e.s.d. in the dihedral angle between two l.s. planes) are estimated using the full covariance matrix. The cell e.s.d.'s are taken into account individually in the estimation of e.s.d.'s in distances, angles and torsion angles; correlations between e.s.d.'s in cell parameters are only used when they are defined by crystal symmetry. An approximate (isotropic) treatment of cell e.s.d.'s is used for estimating e.s.d.'s involving l.s. planes.
Refinement. Refinement of *F*^2^ against ALL reflections. The weighted *R*-factor *wR* and goodness of fit *S* are based on *F*^2^, conventional *R*-factors *R* are based on *F*, with *F* set to zero for negative *F*^2^. The threshold expression of *F*^2^ > σ(*F*^2^) is used only for calculating *R*-factors(gt) *etc*. and is not relevant to the choice of reflections for refinement. *R*-factors based on *F*^2^ are statistically about twice as large as those based on *F*, and *R*- factors based on ALL data will be even larger.

### Fractional atomic coordinates and isotropic or equivalent isotropic displacement parameters (Å^2^)

**Table:**

	*x*	*y*	*z*	*U*_iso_*/*U*_eq_	
C1	0.5931 (9)	0.6363 (3)	1.0284 (7)	0.0268 (10)	
H1	0.7306	0.6466	1.1350	0.032*	
C2	0.3658 (9)	0.6512 (3)	0.7068 (8)	0.0322 (11)	
H2	0.3429	0.6717	0.5839	0.039*	
C3	0.1931 (10)	0.6034 (3)	0.7302 (8)	0.0360 (12)	
H3	0.0550	0.5936	0.6237	0.043*	
C4	0.4230 (9)	0.5883 (3)	1.0503 (7)	0.0264 (10)	
C5	0.4616 (10)	0.5526 (3)	1.2446 (8)	0.0320 (11)	
N1	0.5632 (7)	0.6684 (2)	0.8557 (6)	0.0256 (9)	
N2	0.2205 (7)	0.5714 (2)	0.9018 (7)	0.0334 (10)	
N3	0.2721 (10)	0.5171 (3)	1.2679 (8)	0.0532 (15)	
H3B	0.2850	0.4963	1.3778	0.064*	
H3C	0.1355	0.5147	1.1732	0.064*	
O1	0.6621 (7)	0.5585 (2)	1.3724 (6)	0.0469 (11)	
Zn1	0.79117 (14)	0.7500	0.82766 (12)	0.0244 (2)	
Br1	0.79321 (15)	0.7500	0.49867 (12)	0.0392 (2)	
Br2	1.13911 (12)	0.7500	1.10460 (11)	0.0351 (2)	

### Atomic displacement parameters (Å^2^)

**Table:**

	*U*^11^	*U*^22^	*U*^33^	*U*^12^	*U*^13^	*U*^23^
C1	0.025 (2)	0.029 (3)	0.023 (2)	−0.0022 (18)	0.0017 (19)	0.001 (2)
C2	0.035 (3)	0.035 (3)	0.023 (3)	−0.001 (2)	0.002 (2)	0.002 (2)
C3	0.033 (3)	0.043 (3)	0.026 (3)	−0.011 (2)	−0.001 (2)	0.003 (2)
C4	0.025 (2)	0.026 (2)	0.027 (3)	−0.0027 (19)	0.0062 (19)	0.001 (2)
C5	0.036 (3)	0.032 (3)	0.026 (3)	−0.009 (2)	0.006 (2)	0.001 (2)
N1	0.0247 (18)	0.028 (2)	0.024 (2)	−0.0028 (17)	0.0075 (16)	−0.0010 (17)
N2	0.027 (2)	0.037 (3)	0.033 (2)	−0.0069 (18)	0.0033 (18)	0.001 (2)
N3	0.044 (3)	0.074 (4)	0.034 (3)	−0.028 (3)	0.000 (2)	0.017 (3)
O1	0.043 (2)	0.055 (3)	0.032 (2)	−0.0183 (19)	−0.0068 (17)	0.0147 (19)
Zn1	0.0224 (4)	0.0275 (4)	0.0247 (4)	0.000	0.0088 (3)	0.000
Br1	0.0424 (4)	0.0536 (5)	0.0242 (4)	0.000	0.0138 (3)	0.000
Br2	0.0222 (4)	0.0511 (5)	0.0302 (4)	0.000	0.0049 (3)	0.000

### Geometric parameters (Å, º)

**Table:**

C1—N1	1.338 (6)	C4—C5	1.499 (7)
C1—C4	1.377 (7)	C5—O1	1.226 (6)
C1—H1	0.9300	C5—N3	1.317 (7)
C2—N1	1.331 (7)	N1—Zn1	2.086 (4)
C2—C3	1.388 (8)	N3—H3B	0.8600
C2—H2	0.9300	N3—H3C	0.8600
C3—N2	1.333 (7)	Zn1—N1^i^	2.086 (4)
C3—H3	0.9300	Zn1—Br1	2.3278 (11)
C4—N2	1.345 (6)	Zn1—Br2	2.3283 (11)
			
N1—C1—C4	120.8 (5)	N3—C5—C4	116.8 (5)
N1—C1—H1	119.6	C2—N1—C1	117.3 (4)
C4—C1—H1	119.6	C2—N1—Zn1	120.6 (4)
N1—C2—C3	121.6 (5)	C1—N1—Zn1	121.7 (3)
N1—C2—H2	119.2	C3—N2—C4	116.3 (4)
C3—C2—H2	119.2	C5—N3—H3B	120.0
N2—C3—C2	121.6 (5)	C5—N3—H3C	120.0
N2—C3—H3	119.2	H3B—N3—H3C	120.0
C2—C3—H3	119.2	N1—Zn1—N1^i^	99.5 (2)
N2—C4—C1	122.4 (5)	N1—Zn1—Br1	105.96 (12)
N2—C4—C5	117.7 (4)	N1^i^—Zn1—Br1	105.96 (12)
C1—C4—C5	119.9 (4)	N1—Zn1—Br2	107.88 (12)
O1—C5—N3	123.9 (5)	N1^i^—Zn1—Br2	107.88 (12)
O1—C5—C4	119.3 (5)	Br1—Zn1—Br2	126.45 (4)
			
N1—C2—C3—N2	−1.7 (9)	C4—C1—N1—Zn1	172.0 (4)
N1—C1—C4—N2	−0.1 (8)	C2—C3—N2—C4	0.5 (8)
N1—C1—C4—C5	179.4 (5)	C1—C4—N2—C3	0.4 (8)
N2—C4—C5—O1	168.4 (5)	C5—C4—N2—C3	−179.2 (5)
C1—C4—C5—O1	−11.2 (8)	C2—N1—Zn1—N1^i^	75.8 (4)
N2—C4—C5—N3	−12.6 (8)	C1—N1—Zn1—N1^i^	−97.0 (4)
C1—C4—C5—N3	167.8 (5)	C2—N1—Zn1—Br1	−33.9 (4)
C3—C2—N1—C1	1.9 (8)	C1—N1—Zn1—Br1	153.3 (4)
C3—C2—N1—Zn1	−171.2 (4)	C2—N1—Zn1—Br2	−171.8 (4)
C4—C1—N1—C2	−1.0 (7)	C1—N1—Zn1—Br2	15.4 (4)

Symmetry code: (i) *x*, −*y*+3/2, *z*.

### Hydrogen-bond geometry (Å, º)

**Table:**

*D*—H···*A*	*D*—H	H···*A*	*D*···*A*	*D*—H···*A*
N3—H3*B*···O1^ii^	0.86	2.01	2.869 (7)	175
N3—H3*C*···N2	0.86	2.38	2.732 (7)	105
N3—H3*C*···N2^iii^	0.86	2.54	3.182 (7)	132
C3—H3···O1^iv^	0.93	2.49	3.414 (7)	172

Symmetry codes: (ii) −*x*+1, −*y*+1, −*z*+3; (iii) −*x*, −*y*+1, −*z*+2; (iv) *x*−1, *y*, *z*−1.
